# Cases of Enteritis

**Published:** 1822-10

**Authors:** R. Wade

**Affiliations:** Member of the Royal College of Surgeons, and Apothecary to the Westminster General Dispensary.


ART. V.—*Cases of Enteritis.* By R. WADE, Member of the Royal College of Surgeons, and Apothecary to the Westminster General Dispensary.

I ATTENDED Jane Durnsford, aged thirty-four, residing in Crown-street, St. Giles's, through a mild attack of fever, and had considered her convalescent. On Saturday morning, June 15th, she expressed herself, indeed, as being quite well, although not so strong as before her illness. I was sent for on the Saturday evening, and found her complaining very much of violent pain in the umbilical region, which was attended with constant vomiting and a quick small pulse; she could not bear the slightest pressure on the abdomen ; even the weight of the bed-clothes increased the pain. She had gone out to walk in the morning, had taken a glass of cider, and on her return home was attacked with pain. Eighteen ounces of blood were taken from the arm, a dozen and half of leeches applied to the abdomen, and afterwards warm fomentations. A cathartic powder and enema were then administered. On Sunday morning, the pain had not in the least degree abated: she was again bled, and the application of the leeches were repeated. At twelve o'clock, the patient was seen by Mr. A. C. Hutchison, at her own particular request, who ordered her to be again bled to twenty ounces, and the immediate use of the warm bath; also a large blister to be applied to the abdomen, on her coming out of the bath. It will be sufficient to state, that the remedies usually employed in enteritis were now persevered in, with a due consideration to her situation,—such as a repetition of the bleedings, warm bath, ol. ricini, &c., but with very slight mitigation of the symptoms, until her death, which took place on Wednesday morning at eight o'clock, ninety hours from the commencement of the attack. The warm bath afforded great relief during the time of immersion, so much so that she expressed a wish to endeavour to sleep in it. Her thirst was incessant from the beginning, and most distressing, from the stomach not being able to retain even a tea-spoonful of barley-water.

The abdomen was examined six hours after death, in the presence of Dr. Granville, Mr. Hutchison, and Mr. Smith (a pupil of Mr. Hutchison's), when the following appearances were observed:—The intestinal convolutions were firmly agglutinated by lymph thrown out by the peritoneal coat of the intestines, in a state of high inflammation: this membrane was covered in several places with a thick yellow coat, very similar to the false membrane formed in croup. The peritoneal coat of the spleen and liver exhibited the same kind of appearances, only in a slighter degree. In the lower portion of the ileum, about eight inches from the appendix cœci, a small sphacelated opening, of the size of a pea, was observed. The mucous membrane was highly inflamed, and nearly in a gangrenous state for three or four inches around the portion of intestine that had sloughed. A pint and a half of sero-purulent fluid was found in the cavity of the abdomen, in which were floating flakes of coagulable lymph, similar to those covering the peritoneal coat of the intestines.

The peritoneum lining the abdominal parietes did not exhibit any mark of inflammation.

Samuel Taylor, father of the woman above mentioned, who had attended closely upon his daughter throughout her illness, on the Saturday night after her death, June 22d, was attacked with rigors, which continued during the Sunday, and were accompanied with slight sickness at stomach. The patient slept during the night, with some degree of wakefulness, and paid little attention to those symptoms until the Monday morning, when he awoke, about five o'clock, with a severe pain in the abdomen, which had much increased at nine o'clock, when Mr. Hutchison first saw him. The patient said "the pain was so violent, that he could not live long without some relief:" his pulse was small and quick, and the countenance expressed great anxiety. Mr. Hutchison ordered thirty ounces of blood to be taken from the arm immediately, and fifteen ounces more at twelve o'clock; he was also ordered, at five o'clock P.M., to take a table-spoonful of castor-oil every hour until the bowels began to act. At this time the pain was still severe, but certainly relieved by the bleeding. Twenty ounces more blood were directed to be taken, the warm bath again had recourse to, and a blister to be applied to the abdomen. The next morning, the pain still continuing, the bleeding was repeated to fourteen ounces. For some hours after this last depletion he felt very little pain, but it returned in the evening. The pulse still continuing very quick, ten ounces more blood were taken away the following morning. The pain was not considerable; he had no vomiting during the night, and the bowels had been slightly relieved. The bleeding was repeated to eight ounces; and it is only necessary now to add, that, the bowels continuing to act, from the administration of the ol. ricini, all unfavourable symptoms gradually subsided from this period, and the man is now (July 31st,) in the enjoyment of perfect health.

I understand that Dr. Hamilton, of Edinburgh, has expressed his conviction that peritoneal inflammation is sometimes contagious: whether the facts above related tend to confirm such an opinion, I shall leave the reader to determine. The similarity of the symptoms in both cases struck Mr. Hutchison and myself very forcibly, without either of us entertaining any such opinion as that alluded to. In reference to the result of the two cases, it must be remembered that the woman was in a debilitated state, just recovering from fever, and that the very quick progress of the inflammation, and the rapidity with which disorganization took place, (as illustrated by the dissection above described,) were so strongly impressed upon our minds, that the bleeding was followed up more vigorously in the latter: indeed the man lost as much blood as could, with safety, have been taken from him in so short a time.

*Note.*—After remaining free from pain for a month, Samuel Taylor had a return of peritoneal inflammation, which appeared to have been brought on from drinking too freely of porter, and having taken more exercise than usual. For this attack, which was much milder than the first, he was bled three times: twenty-four ounces of blood were taken at the first bleeding, fourteen ounces at the second, and ten ounces at the last. He had also a blister applied to the abdomen, and is again convalescent. I mention this return of inflammation, as it shows that great susceptibility to inflammation may remain for some time.

*August 8th*, 1822.

---

*Remarks on the above Cases.* By A. Copland Hutchison, Surgeon to the Westminster General Dispensary, &c.

Mr. Wade, the resident apothecary to this institution, is one of the most zealous young surgeons, in his endeavours to acquire professional knowledge, I have yet met with; and it has been at my request that the two cases of enteritis, above related, were drawn up, as I conceive them to be of sufficient interest to be laid before the profession, through the medium of your respectable Journal: first, because, on a comparison of the two cases, there will be seen to have occurred a very marked difference in the result; and this difference, there can be but little doubt, will appear to arise from the very early and large bleedings which were had recourse to in the case of the father, without which no decided case of this too-fatal disease can be expected to be subdued;—secondly, because the similarity of symptoms between the case of the father and that of the daughter were such as to have surprised us still more, had we not heard of the opinion of Dr. Hamilton, of Edinburgh, and very properly alluded to by Mr. Wade;—thirdly, because Mr. W. has given a very accurate account of the *post mortem* examination of the woman, which is always a valuable document to be recorded;—and, lastly, because the postscript shows the liability to a relapse among those who may have suffered from this disease.

After a careful perusal of these cases, short as they are, the practitioner will see that the surest way to subdue this formidable disease is to bleed early, largely, and repeatedly, but gradually to diminish the quantity of blood taken away at each bleeding, accompanied by the other auxiliaries; for, not unfrequently, two hours' delay may occasion the loss of our patient. This may be said to be no new doctrine; but, when striking facts like those before us are constantly coming under our immediate notice, we cannot too often force them upon the particular attention of the junior members of the profession, accompanied, as they are, by the interesting dissection of one of the cases.

That of the woman would seem to show, also, that we ought not to be deterred from an early and free use of the lancet, even in cases of a previously supposed debilitated habit, from whatever cause such debility may have arisen; for it is obvious that, in this case, there was at least no real debility in the vascular system; otherwise we should not have had so extensive disorganization of parts in so short a time, as that which was apparent on the dissection.

The invariable smallness of the pulse in this disease, added to the previous state of debility the poor woman laboured under, would have deterred many much more experienced men in the profession than Mr. Wade from depleting even to the extent he did.

There is yet one more remark I have to make, for the benefit of the young practitioner, which the relation of these cases suggests to me, in order to preclude the possibility of any delay in a free use of the lancet,—namely, the leading diagnostic between enteritis and common colic; and which I hold to be the extreme tenderness of the abdomen to the touch in the former disease: this, coupled with a quick small pulse, and the other symptoms attending enteritis, must point out the real nature of the malady. I notice this because, although bleeding may be useful in colic, there is no necessity for carrying it to the extent we would in the former disease: but this remark is mentioned the more particularly, that we may not be led astray by treating a case of enteritis as a case of colic, which I lately saw, and which proved fatal to the patient.

I may likewise mention it to be my decided opinion, that purgative medicines of any kind should be avoided in enteritis, during the first few hours of attack; and that then the best aperient is the ol. ricini or small doses of the magnesiæ sulphas, until late in the disease, when those of a more powerful nature may be advantageously had recourse to. The oil of croton (croton tiglium,) in its genuine state is said to have a powerful effect as a purgative, when a drop or two, only, has been applied to the tongue, unmixed with any vehicle. If this be so, it proves the great extent of sympathy along the mucous membrane of the whole alimentary canal, and furnishes us with a valuable remedy in a great variety of cases.

